# The Patient-Reported Outcomes Thermometer–5-Item Scale (5T-PROs): Validation of a New Tool for the Quick Assessment of Overall Health Status in Painful Rheumatic Diseases

**DOI:** 10.1155/2018/3496846

**Published:** 2018-10-23

**Authors:** Fausto Salaffi, Marco Di Carlo, Marina Carotti, Sonia Farah

**Affiliations:** ^1^Rheumatological Clinic, Università Politecnica delle Marche, Jesi, Ancona, Italy; ^2^Department of Radiology, Università Politecnica delle Marche, Ancona, Italy

## Abstract

**Objective:**

To investigate the construct validity, reliability (internal consistency and retest reliability), and feasibility of the patient-reported outcomes thermometer–5-item scale (5T-PROs), a new tool to measure overall health status in patients with painful chronic rheumatic diseases such as rheumatoid arthritis (RA), psoriatic arthritis (PsA), axial spondyloarthritis (axialSpA), and fibromyalgia (FM).

**Methods:**

Consecutive patients have been involved in this study. The following analyses were performed to establish the validity of the 5T-PROs: (1) principal component factor analysis was used to identify the presence of a relatively small number of underlying latent factors than can be used to represent relations among sets of many variables; (2) Cronbach's alpha was calculated as an indicator of internal consistency; and (3) Pearson product-moment correlations were conducted to assess the convergent validity. The 5T-PROs was also administered a second time (two weeks after the initial administration) to a subset of sample (*n* = 426) to allow for calculation of test-retest reliability. We used the intraclass correlation coefficient (ICC) as an estimate of test-retest reliability. Additionally, discriminant validity was tested using analysis of variance (ANOVA) with Bonferroni post hoc multiple comparisons, in different disease conditions. Feasibility was analyzed by the time taken in completing the 5T-PROs and the proportion of patients able to complete the 5 item.

**Results:**

1,199 patients (572 with RA, 251 with axialSpA, 150 with PsA, and 226 with FM) were examined. The mean age was 55.7 (standard deviation: 13.1; range: 20 to 80) years. Factor analysis yielded two factors which accounted for 62.54% of the variance of the 5T-PROs. The first factor “Symptom Summary Score” (35.57% of the variance) revealed a good internal consistency (alpha = 0.88); the internal consistency of the second factor “Psychological Summary Score” (26.97% of the variance) was moderate (alpha = 0.69). The reliability of the whole instrument was good (alpha = 0.82). A very high correlation was obtained between Symptom Summary Score and SF-36 PCS and between pain thermometer intensity and SF-36 bodily pain. For all five items and summary scale scores of the SF-36, there was strong evidence that the mean rank of the scores differs significantly between the groups (Kruskal–Wallis tests, *p* < 0.001). Discriminant validity, assessed by comparing the 5T-PRO dimensions in patients with different states of disease activity, showed that the 5T-PROs show moderate association with the presence of comorbidities. It was also noted that it was inversely correlated (*p*=0.01) to years of formal education.

**Conclusion:**

The 5T-PROs is easily administered, reliable and a valid instrument for evaluating the extensive multidimensional impact associated with chronic painful rheumatic conditions.

## 1. Introduction

Rheumatoid arthritis (RA), axial spondyloarthritis (axialSpA), and psoriatic arthritis (PsA) are common chronic painful rheumatic diseases characterized by systemic inflammation, joint destruction, and impairment in physical function and health-related quality of life (HRQoL). Fibromyalgia (FM) is a chronic disease characterized by muscle pain and other multisymptoms such as fatigue, morning stiffness, memory, and mood issues. RA is the most frequent inflammatory rheumatic disease, with a prevalence of 0.5% in the general adult population [1]. Patients with active RA showed to suffer deficits in HRQoL, along with a number of limitations in physical functioning and mental health dimensions: pain, fatigue, and disability are common challenges that may subsequently lead to psychological distress [2]. Furthermore, patients with RA who have significant functional disability have a 3-fold increased risk of mortality compared with that of the general population, and this risk is comparable with that of individuals of the general population in the highest quintile for systolic and diastolic blood pressure, cholesterol level, or pack-years of smoking [3]. AxialSpA has a heterogeneous clinical presentation and does not have a single pathognomonic feature that distinguishes the disease from other conditions with similar symptoms. In daily rheumatological practice, a diagnosis of axialSpA is generally made in patients with chronic back pain on the basis of a combination of symptoms from medical history, physical examination, laboratory investigations, and findings on imaging. Similar to other chronic diseases, axialSpA can affect quality of life, morbidity, mortality, participation in paid and unpaid work, and healthcare costs [4]. PsA is an inflammatory peripheral and/or axial arthritis associated with psoriasis, usually seronegative for rheumatoid factor. In Italy, it has been estimated to be 36% in psoriatic subjects and 0.42% in general population [1]. In addition to the peripheral joint disease, patients with PsA have a debilitating skin disease, and up to 50% may also have spinal disease [5]. Compared to RA and ankylosing spondylitis (AS), there is less information about the burden of illness in PsA [6, 7]. Although considered a benign disease in the majority of cases given in previous reports or in population-based samples [8], clinical cohort studies described PsA as a progressive, disabling disease, particularly when polyarticular peripheral arthritis is present [9]. FM affects approximately 2-3% of the general population (more than 90% of the patients are female), and usually pain is the most important symptom [1, 10]. FM has a deep impact on global well-being [8] and has been found to be associated with high rates of use of healthcare resource and an increased risk of being unable to work [11]. Traditional methods of evaluation, focused on the musculoskeletal system and measures of impairment, may fail to describe the extensive multidimensional issues associated with chronic painful rheumatic conditions. Consideration of HRQoL has become increasingly important on decisions regarding resource allocation, intervention design, and pharmacological treatment with biologic agents of individuals with chronic inflammatory disabling conditions [12, 13]. Improvements in pain, fatigue, physical function, emotional well-being, and patient global ratings of health are often more important and meaningful in disease assessment than improvements in composite disease activity measures [14–16]. The relevance of patients preference is highlighted by the Outcome Measures in Rheumatology (OMERACT) [17, 18], by the American College of Rheumatology (ACR) (http://www.rheumatology.org/Practice/Clinical/Clinical_Support/2015), by the European League Against Rheumatism recommendations (EULAR), by the Group for Research and Assessment of Psoriasis and Psoriatic Arthritis (GRAPPA) [19, 20], by the Assessment in Spondyloarthritis International Society (ASAS) [21], and in the US Food and Drug Administration guidance [22]. All the scientific societies underline the importance of including clinically relevant patient-reported outcomes (PROs) when designing clinical trials in rheumatic diseases [23–26].

The increasing focus on PROs in rheumatology has had the positive effect of giving prominence to the views and experiences of patients [14].

PROs have been implemented globally and have correlated significantly with objective values in rheumatologic diseases and other chronic pathologies (i.e., cancer, asthma, hypertension, heart disease, stroke, psychiatric illness, migraines, and diabetes) [27–29]. Despite the proliferation of tools and the burgeoning theoretical literature devoted to these measurements, no unified approach has been devised for PROs application in clinical practice, and little agreement has been attained about mean this lack of standardization of outcome measures, limiting the usefulness of clinical trial evidence to inform healthcare decisions; moreover, PROs can be difficult to be administered, scored, and interpreted in clinical practice.

Of utmost importance is the graphic presentation that influences the psychometric properties of each instrument. Usually numerical rating scales (NRS) and verbal descriptor scales (VDS) are preferred for older adults, which may be find more difficulties with other types of scales [30]. The thermometer scales, a modified vertical VDS alongside a graphic thermometer, have also been validated as a measure for pain in older adults and are recommended and commonly used in clinical practice in inflammatory arthritis [31].

**Figure 1:**
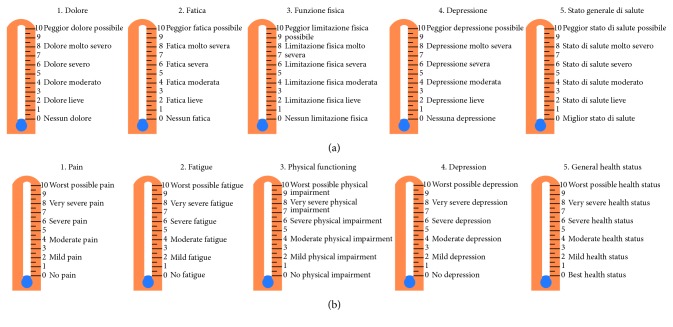
The Italian (a) and English (b) versions of the Patient-Reported Outcomes Thermometer–5-item scale (5T-PROs).

Time constrains usually hinder the evaluation of HRQoL through long and difficult to compute instruments. Thus, we developed the Patient-Reported Outcomes Thermometer–5-item scale (5T-PROs), a simple tool made of 5 “thermometers” combining NRS and VDS (Figure 1), exploring the main domains of HRQoL, namely, pain, fatigue, physical function, depression, and general health status.

The aims of this study were to investigate the construct validity, reliability (internal consistency and retest reliability), and feasibility of this new tool in patients suffering from chronic inflammatory joint diseases and FM.

## 2. Materials and Methods

### 2.1. Study Population

Participants at this study were part of an ongoing longitudinal project measuring rheumatic disease outcomes, started in 2005. This longitudinal project involves consecutive adult patients coming from the Rheumatological Clinic of the Università Politecnica delle Marche, Jesi (Ancona). The study population was represented by patients suffering from RA, PsA, axialSpA, and FM. All the diagnoses were made according to the international criteria for each disease [32–36].

All procedures performed were approved by the institutional review board (Comitato Etico Unico Regionale), and written informed consent for anonymous analysis of data was obtained from all individual participants.

### 2.2. Measurements and Instruments

A comprehensive questionnaire package (including sociodemographic data, disease duration—years since fulfilment of the classification criteria of the disease, quality of life measuring tools, and disease-related variables) was administered to the patients. The sociodemographic variables assessed were age, sex, and level of education (primary; secondary; and high school/university). Furthermore, the presence of comorbidities were assessed using additional questions asking for the presence of nine specific comorbid conditions (hypertension, myocardial infarction, lower extremity arterial disease, major neurological problem, diabetes, gastrointestinal disease, chronic respiratory disease, kidney disease, and poor vision). The algebraic sum of positive responses was calculated for each subject, giving a comorbidity factor with a possible range from 0 to 9.

### 2.3. Disease-Related Characteristics

Disease-related characteristics included the measures for disease activity. The Clinical Disease Activity Index (CDAI) was used to evaluate disease activity in patients with RA [37], the Disease Activity index for PSoriatic Arthritis (DAPSA) was employed for peripheral PsA [38], while the Ankylosing Spondylitis Disease Activity Score C-reactive protein (ASDAS-CRP) was used to assess disease activity in patients with axialSpA [39]. FM was evaluated trough the Fibromyalgia Impact Questionnaire—revised version (FIQ-R) [40].

The CDAI is based on the simple sum of the swollen/tender joint counts-28 joints, along with patient and physician global assessment (PaGA and PhGA, respectively) of disease activity (on a 0–10 VAS scale) [37]. The CDAI result can range from 0 to 76. High disease activity is defined as a CDAI > 22, moderate disease activity with 10 < CDAI ≤ 22, low disease activity 2.8 < CDAI ≤ 10, and remission as a CDAI ≤ 2.8 [41].

DAPSA was adapted from the Disease Activity Index for Reactive Arthritis (DAREA), a score developed and validated to assess reactive arthritis. Developed from a clinical cohort [38] and validated using clinical trial data [42], DAPSA comprises 68 tender and 66 swollen joints count, PaGA, pain (0–10 NRS), and CRP in mg/dl. The final score is the sum of these variables. Recently, DAPSA cutoffs for disease activity states and treatment response have been derived using patient level data from three PsA randomized controlled trials [43]; therefore, this index is now usable and interpretable.

The ASDAS is the first validated disease activity index that considers together self-reported items and objective measures including back pain, duration of morning stiffness, peripheral joint pain and/or swelling, PaGA, and a serologic marker of inflammation (ESR or CPR) [39]. The cutoffs defining the disease activity ranks are as follows: <1.3 inactive disease, ≥1.3 and <2.1 moderate disease activity, ≥2.1 and <3.5 high disease activity, and ≥3.5 for very high disease activity.

The FIQ-R is an updated version of the FIQ [44]. The new version, validated in Italy for its use in patients with FM [45], has 21 items (all based on an 11-point NRS, with 10 being the “worst”) and covers the three domains of function (9 items), overall impact (2 items), and symptoms (10 items). The questions are framed in the context of the previous seven days, and the total maximum score is 100 (higher scores indicating greater disease impact). The FIQ-R score is the sum of the three domain scores: the summed score for the 9-item function domain (range 0–90) is divided by three; the summed score for the 2-item overall impact domain (range 0–20) remains as it is; and the summed score for the 10-item symptom domain (range 0–100) is divided by two.

### 2.4. Health-Related Quality of Life (HRQoL) Assessment

HRQoL was assessed using well-validated generic instruments such as the self-administered SF-36 questionnaire [46] and EuroQoL-5 dimensions (EQ-5D) [47]. The Short-Form 6-dimensions (SF-6D) was estimated from the SF-36 [48].

The 36 items are comprised in the eight scales cover the following health domains: physical functioning (PF), role limitations due to physical function (RP), bodily pain (BP), general health (GH), mental health (MH), role limitations due to emotional health (RE), social functioning (SF), and vitality (VT). One additional item pertains to health transition. The raw scores were encoded and reweighted (items summed and transformed to the eight 0–100 scales, with a final value ranging from 0 = worst health to 100 = best health) [46]. The SF-36 has been validated for use in Italy [49] and can be completed within 15 minutes by the majority of the subjects. Two psychometrical summary measures can be derived from SF-36: the physical and the mental component summary score (PCS and MCS) [46].

EQ-5D is directed to the domains of mobility, self-care, usual activities, pain/discomfort, and anxiety/depression. Each domain has one question, and each question has three levels: one denoting no problems and three denoting severe problems [47]. The Italian population-based values were used to convert patient responses to the health status classifier into a single index which produces scores from 1 to −0.38 [50, 51]. In addition, patients were asked to rate their current health status on a vertical, graduated 20 cm VAS (EQ-5D VAS), ranging from 0 (worst possible health state) to 100 (best possible health status).

Finally, the SF-6D was collected. Derived from the SF-36 [49], SF-6D is focused on six of the eight health domains: PF, role participation (combining RP and RE), SF, BP, MH, and VT. The SF-6D is calculated using a definite scoring function [48] in order to create a weighted index score ranging from 1.0, no difficulty in any dimensions (or perfect health), to 0.296 (severely impaired levels in all dimensions).  Table 1 provides an overview of the HRQoL instruments.

### 2.5. The Patient-Reported Outcomes Thermometer–5-Item-Scale (5T-PROs)

The 5T-PROs is a five-item measure which consists of thermometers with numerals displayed vertically from 0 to 10. It has a broader perspective and better coverage of the domains in the International Classification of Functioning, Disability and Health (ICF), and identified as important by people with rheumatic disorders [52, 53].

Patients rate the five thermometers with a recall of one week: 0 indicates no pain, fatigue, physical impairment, depression and best health status, and 10 indicates worst possible pain, fatigue, physical impairment, depression, and general health status. These five measures afford a simple and rapid administration and increased comprehension and completion rates. The 5T-PROs is a tool that can help both the person and staff to begin a conversation with each other about the wider range of difficulties, together with the services and resources that may be helpful in addressing them. The advantages of this tool are the brevity of the questionnaire, the ease of assessing the results, and its less-stigmatizing format. In this study, we administered the 5T-PROs using a single sheet of paper (Figure 1).

### 2.6. Statistical Analysis

The Kolmogorov–Smirnov test was used to assess distribution of the 5T-PROs, SF-36, EQ-5D, and EQ-6D scores. The interval measurements were normally distributed, and therefore several parametric tests were employed to analyze data. The critical values for significance were set at *p* < 0.05. Following standard guidelines for the evaluation of measurement properties of quality of life instruments [54–56], we tested validity, reliability, and feasibility of 5T-PROs. Construct validity was assessed by performing principal components factor analysis on individual 5T-PROs scales. An eigenvalue criterion of 1.0 was used to select factors, and the results are given in terms of the percentage of variance in the scale score explained by the principal factor. Convergent validity was tested by correlating (Pearson's *r*) the scores of the 5T-PRO subscales with the other measures applied in the study. One-way ANOVA was performed to test for differences. A particular subscale is expected to converge with the scores of those instruments targeting the same construct and to deviate from the scores given by instruments or scales assessing a different one (divergent validity). To investigate a possible influence of patient characteristics, such as age, gender, educational level, and the number of comorbid conditions on the 5T-PROs, the associations between the total score and these features were also analyzed. The internal structure and reliability of the 5T-PROs scales were evaluated by means of internal consistency (Chronbach's alpha coefficient) and test-retest reliability [55]. Chronbach's alpha statistic measures the overall correlation between items within a scale. It ranges from zero to 1, and values equal or greater than 0.80 indicate adequate internal consistency for a scale [57]. Inter-item correlations compares scores on individual items with the total score of the scale. Items with item-total correlations less than 0.4 should be considered as rejects. To evaluate reproducibility, 434 randomly selected patients (189 with RA, 67 with PsA, 45 with axialSpa and 133 with FM) completed the 5T-PROs twice with a time interval of 7 days. The opinions regarding the appropriate interval vary from an hour to a year depending on the task, but a test-retest interval of two to 14 days is common for this type of questionnaire [54]. Reproducibility concerns the degree to which repeated measurements in stable persons provide similar results. Test-retest reliability (reproducibility) was evaluated using intraclass correlation coefficient (ICC) [55], that assesses the correlation of scales at two different measure points. The values of ICC vary from 1 (perfectly reliable) to 0 (totally unreliable), and values above 0.80 were considered as evidence of excellent reliability [56]. The Bland and Altman method was used to quantify agreement, by calculating the mean difference (Mean Δ) between the two measurements and the standard deviation (SD) of this difference [58]. Finally, to assess the patient's acceptance and feasibility of 5T-PROs, the participants filled out an additional questionnaire. The patient's acceptance was established by asking the following questions: (a) is the 5T-PROs easy to use? (b) Is the 5T-PROs format user-friendly? (c) Is the 5T-PROs easy to understand? (d) The 5T-PROs works well (is reliable)? (e) In general, are you satisfied with using the 5T-PROs? Further, feasibility was evaluated by the time taken to complete the 5T-PROs, which was recorded by a research assistant using a stopwatch and the time taken to complete the questionnaire. Finally, we assessed the presence of floor and ceiling effects, by examining the frequency of the highest and lowest possible scores at baseline. Floor effects were considered to be present if more than 15% of the patients had a minimal score at the baseline, and the ceiling effects were considered to be present if 15% of the patients had a maximum baseline score [59]. Data were stored in a FileMaker 7.0 relational database and has been processed with the SPSS 11.0 and MedCalc 17.8 for statistical software packages for Windows XP.

## 3. Results

### 3.1. Demographic and Clinical Data

Of the 1,298 patients enrolled, 1,199 (92.4%) subjects (572 with RA, 251 with axialSpa, 150 with PsA, and 226 with FM) completed the clinical assessment and the questionnaires, ninety-nine (7.6%) were excluded because of incomplete data and nonrespondents were significantly older (*p* < 0.001). The majority of the sample were women with primary or secondary educational level. The respondents' age ranged from 19 to 80 years, with a mean of 55.5 years (SD = 12.2 years). The age and sex distributions of the patients with RA, PsA, axialSpA, and FM were significantly different (*p* < 0.001). The mean (±SD) age was 57.6 ± 14.5 years for RA, 60.4 ± 12.1 years for PsA, 53.1 ± 10.4 years for axialSpA, and 50.7 ± 10.1 years for FM. Slightly more than one quarter of the patients with RA, more than two thirds of the patients with axialSpA, and slightly less than an half of the patients with PsA were male. In FM patients, only 16.4% were male. Mean (±SD) disease duration was similar in PsA and axialSpA (4.6 ± 3.3 and 4.5 ± 3.2 years, respectively), while it was higher (*p*=0.02) in RA (6.7 ± 4.4 years) and in FM (5.9 ± 4.1 years). The educational level among patients with RA was lower than among patients with PsA and axialSpA (*p* < 0.02). Of the 1,199 subjects enrolled, 867 (72.3%) reported one or more medical comorbidities. The frequency of multimorbidity was higher in those subjects classified with PsA followed by that of those classified as RA, axialSpA, and with FM. The most prevalent combinations were with arterial hypertension (10.8%), hypercholesterolemia (7.9%), digestive diseases (6.3%), cardiologic diseases (5.4%), and diabetes mellitus (3.5%). The demographic and disease characteristics of patients enrolled in the study are shown in Table 2.

### 3.2. Disease Activity and Health-Related Quality of Life

Table 3 provides statistics summaries: the mean and SD for each of the aspects of health status covered by the SF-36, EQ-5D, SF-6D, and 5T-PROs and by the disease activity indices for the different diagnostic groups.

### 3.3. Score Distribution

**Figure 2:**
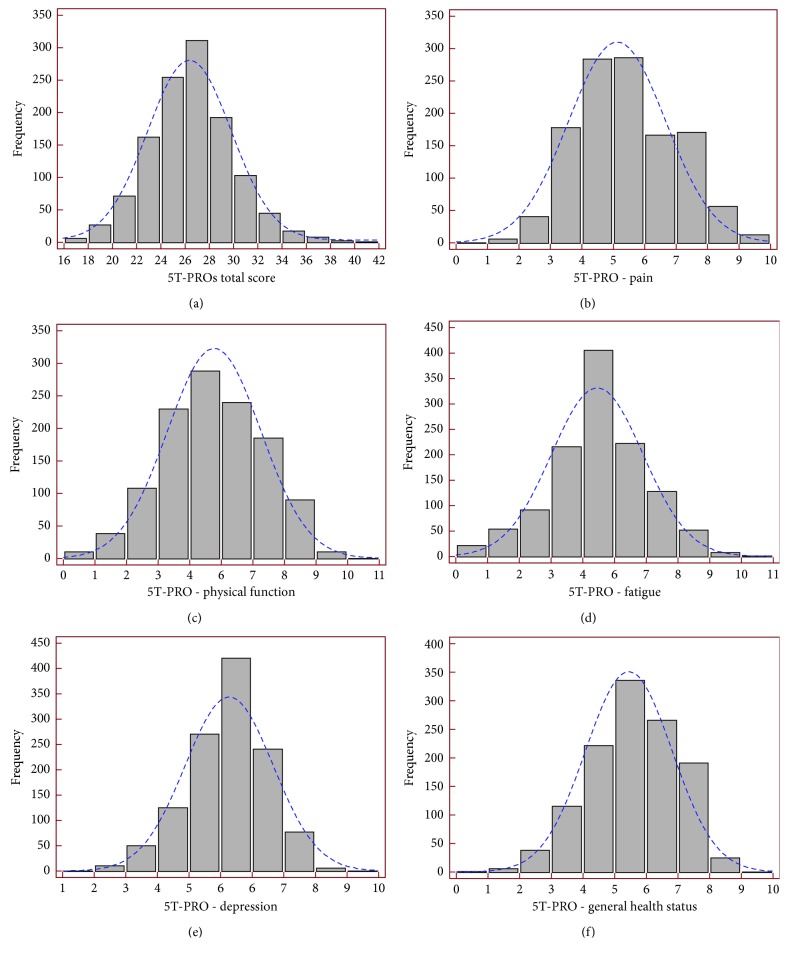
Distribution of the Patient-Reported Outcomes Thermometer–5-item scale (5T-PROs): Total score (a) and the five domains (b)–(f) in 1,199 patients with chronic rheumatic diseases. Floor effect is noted by the percentage of values at 0 for each item. Ceiling effect is indicated by the percentage of values at 100. For descriptive purposes, normal distribution, displayed as vertical lines, has been superimposed on the histogram.

The number of patients receiving floor or ceiling effects was low for the 5T-PROs subscales, with one exception. The 5T-PROs total score distribution is described in Table 3.  Figure 2 presents the estimates of central tendency and distributions for 5T-PROs total score and domains. The bar on the left of each graph represents the number of subjects with a score of 0 (floor effect), and the bar on the right represents the number of subjects with a maximum possible score (ceiling effect).

**Figure 3:**
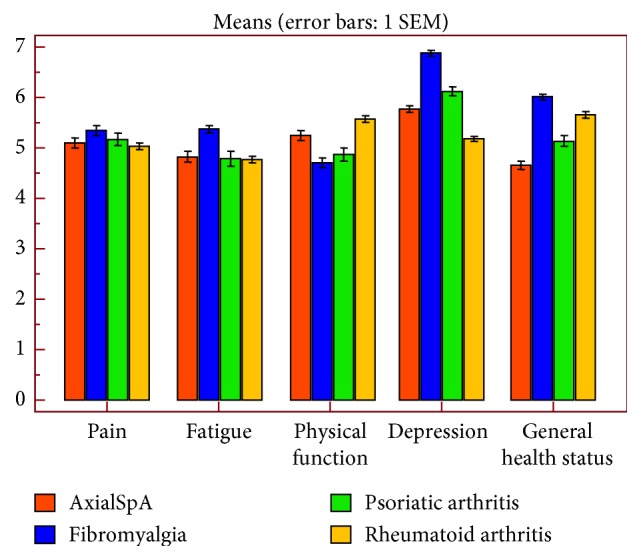
The Patient-Reported Outcomes Thermometer–5-item scale (5T-PROs) domains in the four rheumatic disorders. Bars to show mean and SEM of pain, physical function, fatigue, depression, and global health status in patients with axial spondyloarthritis, psoriatic arthritis, fibromyalgia, and rheumatoid arthritis.

All the eight health concepts of the SF-36 and those of utility scores (EQ-5D, EQ-VAS and SF-6D) were impaired in the four categories of rheumatic disorders (Table 4).  Figure 3 compares the scores in each domain of the 5T-PROs in the different diseases. Overall, the dimensions typically affected were depression and general global health; the disease with the worst HRQoL for those dimensions was FM. The mean depression score of FM patients was 6.87 (SD = 0.77). The mean 5T-PROs global health status of FM patients was 6.00 (SD = 0.98). Regarding the HRQoL dimensions involving physical function, patients with RA score generally higher than the FM patients (Figure 3).

### 3.4. Construct Validity

Factor analysis was carried out to examine the factorial structure of the Italian version of the 5T-PROs. Items were accepted on the final factors if they had a loading of more than 0.50 on the corresponding factor. The analysis revealed a two-factor solution (eigenvalues 1.819 and 1.308) (Table 5). The first factor, namely, the 5T-PRO physical summary score, accounted for the 35.57% of the explained variance and represents the patients rating of the grade of pain, disability, and global health perception in different areas of daily life he or she is suffering from. The second factor, the 5T-PROs psychological summary score, accounted for the 26.97% of the explained variance, representing the patients rating of his medium emotional complaints.

Table 5 shows the loading of each question after varimax rotation with Kaiser normalization on the two factors. Each factor loading represents the correlation between that item and the underlying factor. Both the two dimensions of 5T-PROs (physical and psychological summary scores) correlated significantly with each other (*r* = 0.548; *p* < 0.001).

### 3.5. Internal Reliability

Cronbach's alpha was 0.81 for the 5T-PROs. Both subscales of the 5T-PROs showed satisfying to good internal consistency. Cronbach's alpha was 0.81 for the first factor (physical summary score) and 0.85 for the second factor (psychological summary score). Item-total correlations, which are another measure of internal consistency, compare scores on individual items with the total score of the scale. Items with item-total correlations less than 0.4 should be considered for rejection. In our analysis, item-total correlations for the subscales were moderate up to high (Table 6).

### 3.6. Reproducibility

Equivalence between the two administrations of the 5T-PROs was measured by calculating single-measurement ICCs between corresponding scales. The ICCs ranged from 0.822 (“fatigue” domain) to 0.913 (“general health status” domain) for all the domains in the 5T-PRO, indicating excellent agreement between two administrations (Table 7). All scales met Cicchetti's criterion of 0.75 [60].

**Figure 4:**
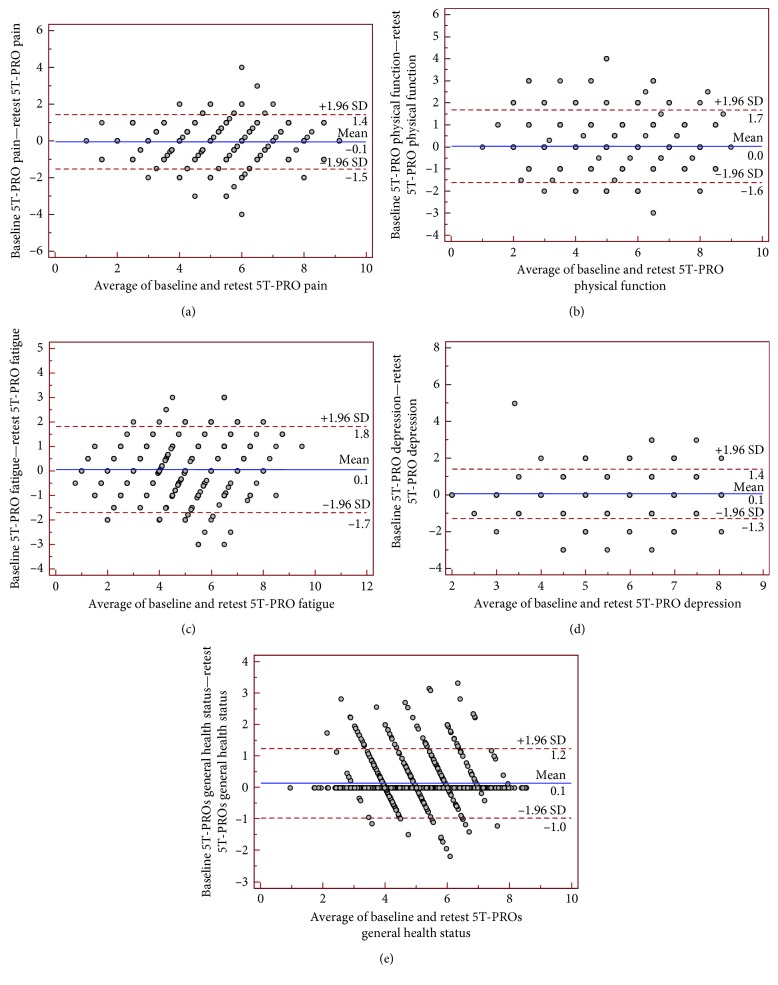
Bland and Altman plot of repeatability with the differences in the Patient-Reported Outcomes Thermometer–5-item scale (5T-PROs). Subscales values ((a) pain; (b) physical function; (c) fatigue; (d) depression; (e) general health status) plotted against average values for the 434 randomly selected patients (189 with rheumatoid arthritis, 67 with psoriatic arthritis, 45 with axial spondyloarthritis, and 133 with fibromyalgia) who completed the 5T-PROs twice with a time interval of 7 days. Ninety-five percent of the differences against the means were less than two standard deviations (SD; dotted lines).

Agreement between scores was also illustrated by Bland and Altman plots, in which the difference between scores was plotted on the *y*-axis against the average of scores on the *x*-axis. According to Bland and Altman analysis, there was no systematic error in scores of 5T-PROs (Figure 4).

### 3.7. Convergent Validity

In testing for convergent validity between instruments, we found that correlation coefficients for the comparable dimension of the 5T-PROs and the SF-36 questionnaires ranged from 0.049 to 0.626. Generally, higher significant correlations were seen when comparing 5T-PROs scales to SF-36 scales with a high ability to measure similar health concept (convergent construct validity) (Table 8).

Of special interest are the correlations between the 5T-PROs total score and disease activity indices such as CDAI for RA (*r* = −0.709; *p* < 0.001), DAPSA for PsA (*r* = 0.479; *p* < 0.001), and ASDAS-CRP for axialSpA (*r* = 0.549; *p* < 0.001), and between 5T-PROs total score and FIQ-R for FM (*r* = 0.722; *p* < 0.0001). Positive correlations between the total 5T-PROs score were also found with the number of comorbidities (*r* = 0.93; *p*=0.001) and educational level (*r* = 101; *p*=0.001).

### 3.8. Acceptance and Feasibility of 5T-PROs

The mean time to complete the 5T-PROs was 3.1 ± 1.3 minutes (range 2.2–9.3 minutes). Overall, the 5T-PROs was correctly completed by most respondents. Less than 3% of each of the 5T-PROs questions had missing values. In subjects who expressed a preference, the majority rated that the tool was easy to fulfill. Patients' preference was not related to sex or age.

## 4. Discussion

There is growing recognition of the importance of placing patients at the center of healthcare by developing patient-centered care models and integrating patient-valued outcomes into shared decision-making [61]. PROs contribute fundamental information from the point of view of people that live with a chronic painful disease, and its treatments about the status of or a change in their physical, emotional, and social health outcomes [62] have become increasingly popular as measurement instruments in epidemiological studies.

In RA and SpA, three PROs have been included within the American College of Rheumatology core set of outcome measures recommended for use in randomized clinical trials [63] as a part of the OMERACT PsA Core Domain Set [64] and the International Classification of Functioning, Disability and Health Core Set for AS [65] and clinical care including global ratings of disease activity or health, pain, and physical function; more recently, fatigue and emotional distress also has been recommended for inclusion [25, 63, 66, 67].

Pain is the most prominent symptom in the majority of the subjects with chronic musculoskeletal conditions, and is the most important determinant of disability. Accurate assessment of pain intensity, which is a necessary prerequisite to rational choice of medical and rehabilitation interventions, represents a clinically challenging proposition. In recent years, several studies began to address the psychometric properties of a variety of pain intensity assessment scales. Among them, the pain thermometer, a modified vertical VDS alongside a graphic thermometer, has also been validated as a measure for pain in older adults [68]. A growing number of studies showed that pain is the strongest factor driving the patient global assessment in inflammatory rheumatic diseases [68, 69].

The pain, and the consequent physical disability, affects social functioning and mental health, further diminishing the patient's quality of life [70].

The second factor considered is the fatigue, a frequent symptom in several inflammatory diseases. Overman et al. evaluating 30 rheumatic diseases showed that severe fatigue is a widespread and highly prevalent problem across rheumatic diseases [71] exacerbating pain and depressive symptoms that have a devastating effect on daily functioning and overall well-being [72]. Therefore, addressing the management of fatigue may also improve a larger cluster of symptoms, like decreased strength accompanied by a feeling of weariness, sleepiness, and irritability [73, 74]. In rheumatic diseases, the association between fatigue and pain has been well established [75, 76]. In RA, it is an important outcome to evaluate according to OMERACT [77], and it has been associated with the Disease Activity Score-28 joints (DAS28) and the CDAI. In SpA, fatigue is part of the Bath Ankylosing Spondylitis Disease Activity Index (BASDAI), and it is more strongly related to the disease process than patient-related variables [78]. Furthermore, fatigue is common in various rheumatic conditions, although most publications concerned fatigue in RA or SpA [79]. In these pathologies, the frequency of fatigue ranges from 42% to 80% depending on the definition and methods of assessment [76, 80, 81]. For 75% of patients with AS and 50% of those with RA, fatigue was considered severe [76, 82]. The fatigue experienced by people with AS is reported to be related to disease activity, poorer functional ability, pain, stiffness, depression, lower global well-being, impaired working, and enthesitis [83–90]. Severe fatigue, more than just being tired, is a typical feature also of FM, affecting up to 4 out of 5 subjects. For patients with FM, fatigue is a complicated, multifactorial, and persistent, as evidenced by longitudinal studies over 5 years [71, 91]. Patients with FM may experience fatigue physically (lack of energy and physical exhaustion), emotionally (lack of motivation), cognitively (inability to think or concentrate), or via the symptom's impact on virtually any aspect of living, such as the ability to work, meet family needs, or engage in social activities [92].

Depression is more common in RA than in the general population and has been associated with increased pain, fatigue, reduced HRQoL, increased levels of physical disability, affected patient global assessment, and increased healthcare costs [93–102]. Depressed RA patients have poorer long-term outcomes and more comorbidities [103] and increased mortality levels [104]. However, prevalence estimates for depression in RA range between 9.5% [105] and 41.5% [106], making it difficult to establish the likely impact of depression in this patient group. Recently, psychological disorders such as depression have been frequently reported in patients with axialSpA [107]. Depression was associated with clinically significantly worse physical functioning, measured with both the Health Assessment Questionnaire and the SF-36 in RA [108]. Moussavi et al. found that the combination of depression and arthritis was cross-sectionally correlated with lower health status, more than depression alone, arthritis alone, or 2 somatic conditions [109]. Morris et al. showed that depression and even intermittent depression over time was associated with low self-reported health status and disability after 18 years [110]. Anxiety and depression are major factors affecting a HRQoL of patients with FM, and the associated symptoms (inability to concentrate, loss of motivation, disturbed sleep, fatigue, and pessimistic mood) may affect their response to treatment and rehabilitation programs [12–111]. Furthermore, negative mood seems to contribute to the persistence of chronic widespread pain [112].

A major use of health measurement scales is to detect health status changes over time, and a priority may be efficiency, i.e., responses achieved using the shortest possible questionnaire [113, 114]. A shorter version would further enhance its applicability in epidemiologic studies, clinical trials, and daily clinical practice [115] since short questionnaires result in improved patient compliance and response rates and are thought to improve the quality of the response [116–118].

Developing an instrument is an ongoing consuming process; effort, costs, and testing validity arise not from a single powerful experiment, but from a series of converging experiments [54]. The current study was conducted to examine and to validate the psychometric properties of the 5T-PROs, a five-item measure which consists of “thermometers” with numerals displayed vertically from 0 to 10, within a population of patients with RA, PsA, axialSpA, and FM. There were three main findings, the first regarding construct validity, specifically, factorial analysis in patients with rheumatic diseases generally supports the factorial validity of the 5T-PROs and suggests the use of separate scores for physical and psychological aspects. Altogether, they explain 69.2% of the variance of the entire questionnaire and indicate high construct validity. The second finding was that the final version of the 5T-PROs showed very good internal consistency; the Cronbach's alpha ranged from 0.74 to 0.91, and this indicates that the items measure the same general construct; and that the tool is stable. In addition, the 5T-PROs showed excellent test-retest reliability, with ICC ranging from 0.83 to 0.96. Our third finding concerned the convergent validity, in particular, the 5T-PROs total score was significantly associated with the physical and mental component scores of the SF-36 and clinical measures, and in fact satisfactory significant correlations were found between the PCS score and most of the 5T-PROs domains, especially mobility level, walking and bending, and pain.

This study has a number of strengths, including the use of a large sample of treatment-seeking individuals with rheumatic diseases. However, the study also has limitations; the main concern is that this study did not provide evidence for responsiveness to change or other psychometric tests. Secondly, criterion validity cannot be assessed because there is no previously accepted “gold standard” instrument for measuring the extensive multidimensional impact associated with chronic rheumatic conditions. Nevertheless, this study represents a structured and carefully conducted approach to validate the 5T-PROs in a large number of sample patients with RD. Finally, patients were recruited from tertiary center, and the results might not be generalizable to patients with chronic painful rheumatic disorders treated by a general practitioner or in small practices.

## 5. Conclusion

The present study is an initial step in evaluating psychometric properties of a new instrument to measure the multidimensional impact on patients with chronic rheumatic conditions. The 5T-PROs demonstrated to be feasible and easy to be administered, with reasonably good scale internal validity, reliability, and external validity in the primary setting. It covers most important areas of HRQoL, rarely assessed as primary end-point in studies and in the everyday clinical practice; the 5T-PROs might help clinicians with substantial advantages to assess fundamental health features in patients suffering from chronic painful diseases. However, its sensitivity to change needs still to be studied.

## Figures and Tables

**Table 1:** Overview of the health-related quality of life assessment instruments.

Instrument	Description	Scale
EQ-5D domains^*∗*^	(i) Subject report, addressing 5 questions:	0-1 points
	(a) Mobility	(Worst to best)
	(b) Self-care	
	(c) Usual activities	
	(d) Pain/discomfort	
	(e) Anxiety/depression	

EQ-5D VAS	(i) Vertical 20 cm used to score the patient's health perception	100 representing the best and 0 the worst health

SF-36 domains^*∗*^	(i) Patient report, 36 items	0–100 mm
	(a) Physical functioning	(Worst to best)
	(b) Role-Physical	
	(c) Bodily Pain	
	(d) General health	
	(e) Vitality	
	(f) Role-emotional	
	(g) Social functioning	
	(h) Mental health	

SF-36 PCS and MCS scores	(i) calculated based upon domain scores	Normative value: mean = 50, SD = 10

SF-6D^*∗*^	(i) Patient report, 11 items	0-1 points
	(a) Physical functioning	
	(b) Role participation (RP and RE)	
	(c) Bodily pain	
	(d) Vitality	
	(e) Mental health	

^*∗*^Based on transformed scale scores. Abbreviations: HRQoL = health-related quality of life; MCS = mental component summary; PCS = physical component summary; SF-36 = 36-Item Short-Form Health Survey version 2; EQ-5D = EuroQol-five dimensions; SF-6D = Short-Form-six dimensions.

**Table 2:** Characteristics of patients with rheumatoid arthritis (RA), psoriatic arthritis (PsA), axial spondyloarthritis (AxialSpA), and fibromyalgia (FM).

	RA (*n* = 572)	PsA (*n* = 150)	AxialSpA (*n* = 251)	FM (*n* = 226)
Women (*n*, %)	412 (72.0)	102 (68.0)	99 (39,8)	189 (83.6)
Age, years (mean (±SD))	57.6 (14.5)	60.4 (12.1)	53.1 (10.4)	50.7 (10.1)
Disease duration, years (mean (±SD))	6.7 (4.4)	4.6 (3.3)	4.5 (3.2)	5.9 (4.1)
Educational level, years (mean (±SD))	11.3 (3.6)	8.5 (3.5)	8.6 (3.7)	9.2 (3.8)
Comorbid conditions, *n* (%)				
(i) None	161 (28.1)	25 (16.6)	75 (29.8)	71 (31.4)
(ii) 1	98 (17.1)	39 (26.0)	108 (43.0)	99 (43.8)
(iii) 2	255 (44.6)	45 (30.0)	48 (19.2)	38 (16.8)
(iv) 3 or more	58 (10.1)	41 (27.3)	20 (7.9)	18 (8.0)

**Table 3:** Distribution analysis of the Patient-Reported Outcomes Thermometer–5-item scale (5T-PROs) total score.

5T-PROs total score	
Lowest value	16.38
Highest value	40.99
Arithmetic mean	26.46
95% CI for the mean	26.27 to 26.66
Median	26.50
95% CI for the median	26.21 to 26.60
Variance	11.91
Standard deviation	3.45
Relative standard deviation	0.13 (13.04%)
Standard error of the mean	0.099
Coefficient of Skewness	0.23 (*P*=0.0010)
Coefficient of Kurtosis	0.57 (*P*=0.0010)
Kolmogorov–Smirnov test for normal distribution	Accept normality (*P*=0.097)

**Table 4:** Summary statistics table of the 36-Item Short-Form Health Survey (SF-36) subscales, of the utility questionnaires, of the Patient-Reported Outcomes Thermometer–5-item scale (5T-PROs) subscales, and of the disease activity indices.

	RA (*n* = 572)		PsA (*n* = 150)		AxialSpA (*n* = 251)		FM (*n* = 226)	
	Mean	SD	Mean	SD	Mean	SD	Mean	SD
SF-36 subscales								
BP	28.63	16.33	38.19	19.04	44.29	17.47	35.56	9.69
RP	29.19	14.86	32.58	23.26	38.42	28.17	38.81	17.24
GH	43.60	19.46	45.69	18.18	47.31	20.96	34.41	11.09
PF	39.14	19.83	46.69	21.31	52.13	20.24	49.96	17.35
MH	49.05	22.79	49.46	20.36	53.55	20.95	36.91	13.32
RE	36.25	40.83	33.30	36.02	43.09	30.54	36.86	23.99
SF	46.16	20.81	48.80	22.21	52.02	19.49	39.64	13.82
VT	43.63	17.30	47.86	17.29	48.10	17.68	38.51	11.81
SF-36 MCS	44.74	12.23	41.23	11.33	40.75	10.18	32.12	7.50
SF-36 PCS	30.64	6.20	34.18	6.71	36.88	8.12	38.85	4.78

Utility questionnaires								
SF-6D	0.56	0.07	0.60	0.07	0.62	0.07	0.56	0.05
EQ-5D	0.43	0.14	0.51	0.14	0.54	0.13	0.45	0.11

5T-PROs								
5T-PROs pain	5.03	1.57	5.17	1.46	5.09	1.58	5.34	1.448
5T-PROs fatigue	4.77	1.60	4.78	1.72	4.82	1.77	5.37	1.06
5T-PROs physical function	5.57	1.62	4.87	1.56	5.23	1.56	4.70	1.55
5T-PROs depression	5.18	1.17	6.12	1.09	5.76	1.07	6.87	0.77
5T-PROs general health status	5.65	1.39	5.13	1.29	4.65	1.26	6.00	0.92
5T-PROs total score	26.23	3.51	26.08	3.24	25.57	3.08	28.31	3.16

Disease activity indices								
CDAI	23.81	7.84						
DAPSA			28.04	10.36				
ASDAS-CRP					2.50	1.13		
FIQ-R							50.01	16.0

Abbreviations: RA = rheumatoid arthritis; PsA = psoriatic arthritis; AxialSpA = axial spondyloarthritis; FM = fibromyalgia; SF-36 = 36-Item Short-Form Health Survey; BP = bodily pain; RP = role limitations due to physical function; GH = general health; PF = physical functioning; MH = mental health; RE = role limitations due to emotional health; SF = social functioning; VT = vitality; MCS = mental component summary score; PCS = physical component summary score; SF-6D = Short-Form 6-dimensions; EQ-5D = EuroQoL-5 dimensions; CDAI = Clinical Disease Activity Index; DAPSA = Disease Activity index for PSoriatic Arthritis; ASDAS-CRP = Ankylosing Spondylitis Disease Activity Score C-reactive protein; FIQ-R = Fibromyalgia Impact Questionnaire Revised Version.

**Table 5:** Principal component analysis—total variance explained.

Component	Initial eigenvalues			Extraction sums of squared loadings			Rotation sums of squared loadings		
	Total	% of variance	Cumulative %	Total	% of variance	Cumulative %	Total	% of variance	Cumulative %
1	1.819	36.373	36.373	1.819	36.373	36.373	1.778	35.569	35.569
2	1.308	26.169	62.542	1.308	26.169	62.542	1.349	26.973	62.542
3	0.903	18.068	80.610						
4	0.568	11.359	91.969						
5	0.402	8.031	100.000						

Extraction method: principal component analysis.

**Table 6:** Principal component analysis—rotated component matrix.

Rotated component matrix^a^		
5T-PROs	Component	
	Factor 1 physical component	Factor 2 psychological component
5T-PROs pain	0.865	0.112
5T-PROs fatigue	0.158	0.755
5T-PROs function	0.823	−0.155
5T-PROs depression	−0.053	0.845
5T-PROs general health status	0.571	0.165

Extraction method: principal component analysis. Rotation method: varimax with Kaiser normalization. Abbreviation: 5T-PROs = Patient-Reported Outcomes Thermometer–5-item scale.

**Table 7:** Agreement between the Patient-Reported Outcomes Thermometer–5-item-scale (5T-PROs) scores assessed by intraclass correlation coefficient (ICC).

5T-PROs	Intraclass correlation coefficient	95% Confidence Interval
5T-PROs pain	0.871	0.857 to 0.885
5T-PROs fatigue	0.822	0.799 to 0.842
5T-PROs function	0.871	0.856 to 0.885
5T-PROs depression	0.844	0.826 to 0.861
5T-PROs general health status	0.913	0.896 to 0.927

**Table 8:** Convergent construct validity analysis: correlation matrix of the Patient-Reported Outcomes Thermometer–5-item scale (5T-PROs) component scores and their components versus the eight SF-36 subscales.

	BP	GH	PF	RP	RE	MH	SF	VT	5T-PROs depression	5T-PROs fatigue	5T-PROs physical function	5T-PROs global health
GH	0.164											
	<0.001											
PF	0.392	0.289										
	<0.001	<0.001										
RP	0.251	0.163	0.425									
	<0.001	<0.001	<0.001									
RE	0.240	0.308	0.313	0.311								
	<0.001	<0.001	<0.001	<0.001								
	1199	1199	1199									
MH	0.184	0.479	0.309	0.124	0.411							
	<0.001	<0.001	<0.001	<0.001	<0.001							
SF	0.218	0.254	0.295	0.101	0.252	0.460						
	<0.001	<0.001	<0.001	<0.001	<0.001	<0.001						
VT	0.206	0.395	0.346	0.178	0.373	0.636	0.357					
	<0.001	<0.001	<0.001	<0.001	<0.001	<0.001	<0.001					
5T-PROs depression	0.049	−0.352	−0.054	0.058	−0.322	−0.626	−0.377	−0.507				
	0.088	<0.001	0.061	0.045	<0.001	<0.001	<0.001	<0.001				
5T-PROs fatigue	0.148	0.258	0.310	0.211	0.275	0.385	0.152	0.679	−0.272			
	<0.001	<0.001	<0.001	<0.001	<0.001	<0.001	<0.001	<0.001	<0.001			
5T-PROs physical function	−0.039	0.124	−0.072	0.028	0.099	0.134	0.071	0.141	−0.204	0.132		
	0.174	<0.001	0.012	0.333	0.001	<0.001	0.013	<0.001	<0.001	<0.001		
5T- PROs global health	−0.480	−0.432	−0.632	−0.260	−0.377	−0.568	−0.443	−0.462	0.342	−0.268	−0.109	
	<0.001	<0.001	<0.001	<0.001	<0.001	<0.001	<0.001	<0.001	<0.001	<0.001	<0.001	
5T-PROs pain	0.101	0.134	0.202	0.147	0.154	0.151	0.067	0.150	−0.085	0.242	0.531	−0.195
	<0.001	<0.001	<0.001	<0.001	<0.001	<0.001	0.020	<0.001	0.003	<0.001	<0.001	<0.001
